# Advanced diagnostic imaging utilization during emergency department visits in the United States: A predictive modeling study for emergency department triage

**DOI:** 10.1371/journal.pone.0214905

**Published:** 2019-04-09

**Authors:** Xingyu Zhang, Joyce Kim, Rachel E. Patzer, Stephen R. Pitts, Falgun H. Chokshi, Justin D. Schrager

**Affiliations:** 1 University of Michigan School of Nursing, Applied Biostatics Laboratory, Ann Arbor, MI, United States of America; 2 Department of Surgery, Emory University School of Medicine, Atlanta, GA, United States of America; 3 Department of Internal Medicine, Emory University School of Medicine, Atlanta, GA, United States of America; 4 Department of Epidemiology, Rollins School of Public Health, Atlanta, GA, United States of America; 5 Health Services Research Center, Emory University School of Medicine, Atlanta, GA, United States of America; 6 Department of Emergency Medicine, Emory University School of Medicine, Atlanta, GA, United States of America; 7 Department of Radiology & Imaging Sciences, Emory University School of Medicine, Atlanta, GA, United States of America; 8 Department of Biomedical Informatics, Emory University School of Medicine, Atlanta, GA, United States of America; University of Rochester, UNITED STATES

## Abstract

**Background:**

Emergency department (ED) crowding is associated with negative health outcomes, patient dissatisfaction, and longer length of stay (LOS). The addition of advanced diagnostic imaging (ADI), namely CT, ultrasound (U/S), and MRI to ED encounter work up is a predictor of longer length of stay. Earlier and improved prediction of patients’ need for advanced imaging may improve overall ED efficiency. The aim of the study was to detect the association between ADI utilization and the structured and unstructured information immediately available during ED triage, and to develop and validate models to predict utilization of ADI during an ED encounter.

**Methods:**

We used the United States National Hospital Ambulatory Medical Care Survey data from 2009 to 2014 to examine which sociodemographic and clinical factors immediately available at ED triage were associated with the utilization of CT, U/S, MRI, and multiple ADI during a patient’s ED stay. We used natural language processing (NLP) topic modeling to incorporate free-text reason for visit data available at time of ED triage in addition to other structured patient data to predict the use of ADI using multivariable logistic regression models.

**Results:**

Among the 139,150 adult ED visits from a national probability sample of hospitals across the U.S, 21.9% resulted in ADI use, including 16.8% who had a CT, 3.6% who had an ultrasound, 0.4% who had an MRI, and 1.2% of the population who had multiple types of ADI. The c-statistic of the predictive models was greater than or equal to 0.78 for all imaging outcomes, and the addition of text-based reason for visit information improved the accuracy of all predictive models.

**Conclusions:**

Patient information immediately available during ED triage can accurately predict the eventual use of advanced diagnostic imaging during an ED visit. Such models have the potential to be incorporated into the ED triage workflow in order to more rapidly identify patients who may require advanced imaging during their ED stay and assist with medical decision-making.

## Introduction

Emergency department (ED) crowding is a well-recognized problem in the United States [1–3]. Problems associated with ED crowding have been extensively documented: longer wait time and length of stay (LOS) during ED visit; staff and patient dissatisfaction; higher hospital costs; and negative patient outcomes [4–8]. As a result, many emergency departments are moving toward physician triage models in which physicians perform rapid evaluations to expedite work-ups and dispositions while patients are still in the waiting area. This method has shown promise—with studies demonstrating decreased LOS and decreased number of patients who leave without being seen [9–12]. Algorithmic clinical decision support, specifically predictive analytics, may be of benefit in this clinical setting[13]; however its use has not been sufficiently described or tested.

The decision by an ED provider to pursue advanced diagnostic imaging (ADI) studies during an ED visit is a major contributor to increased ED LOS [14, 15], and ADI use in the ED has been increasing for more than a decade [16]. The median LOS for ED patients with ADI is 114 minutes longer than those without ADI [17]. This increased LOS can be attributed to clinical factors—such as the amount of time it takes to obtain and interpret a CT scan—and diagnostic factors—such as the time it takes to clinically evaluate a patient and decide if they will need ADI. Early prediction of eventual ADI use has the potential to shorten diagnostic time. To date, research has not examined the role of a predictive model that can use information immediately available to a triage provider upon patient arrival (e.g. patient demographics, vitals, medical history, and the patient’s own descriptions of their reason for visit) to estimate the probability that the patient will undergo ADI during their ED visit. Such a predictive model—if implemented and tested in the clinical setting perhaps as an adjunct to the Electronic Health Record (EHR) or as a standalone program—could support clinicians in making rapid, informed decisions regarding ADI. Furthermore, few studies have utilized the important information that exists within the free-text reason for visit that patient’s give on arrival to the ED to make predictions regarding processes and outcomes in the ED [18, 19]. This free-text reason for visit information can be utilized via natural language processing (NLP), a method through which text data can be extracted and processed for analysis and has been shown to improve models related to health outcomes [20–23].

In a nationally representative sample, we examined patient information that would readily be available during the ED triage process, including free-text reason for visit, to develop predictive models for ADI use including computed tomography (CT), ultrasound (US), and magnetic resonance imaging (MRI) during the ED encounter.

## Materials and methods

### Study population

This study is a secondary analysis of data collected from the 2009–2014 National Hospital Ambulatory Medical Care Survey ED Subfile (NHAMCS-ED)[24–26], a multistage, stratified probability sample of ED visits in the United States administered by the National Center for Health Statistics, a branch of the Centers for Disease Control and Prevention. The NHAMCS-ED sample is collected during a random 4-week period each year. Study staff visit approximately 300 hospital-based EDs, which are randomly selected from approximately 1,900 geographically defined areas covering all 50 States and the District of Columbia. A standardized form and protocol are utilized to abstract data from approximately 100 patient charts per ED. Details of the survey methodology are available from the National Center for Health Statistics [25, 26]. A total of 179,036 patient visits were included in the survey datasets from 2009 to 2014. After excluding pediatric visits (n = 39,886), 139,150 (77.7%) adult patients (≥18 years old) visits remained for analysis.

### Study variables

#### Outcome variables

The primary outcome variables for this study were the eventual use of advanced diagnostic imaging during an ED visit. ADI categories were analyzed independently and include any ADI, CT only, US only, MRI only, or multiple ADI use.

#### Structured variables

Structured covariates included only those that would be immediately available at the time of ED triage: sex, age category, race/ethnicity, type of residence (private residence, nursing home, homeless, or other), source of payment (using the NHAMCS algorithm to classify multiple payers), whether the patient arrived via ambulance, arrival day and time, initial vital signs (body temperature, heart rate, respiratory rate, blood pressure, pulse oximetry), pain scale, whether the patient arrived on oxygen, whether the patient had used the ED within the past 72 hours, the episode of care (initial vs. follow-up visit to the ED for the presenting problem), past medical history (cancer, cerebrovascular disease, chronic obstructive pulmonary disease, condition requiring dialysis, congestive heart failure, dementia, diabetes, myocardial infarction, pulmonary embolism, and HIV), whether the visit was related to an injury, poisoning, or adverse effect of medical treatment (and if related to injury/poisoning, whether it was self-inflicted, related to assault, unintentional, or unknown), and visit acuity at triage (triage level which by convention is changed to a 5 point system by NHAMCS researchers when EDs use a 3-point or 4-point acuity scale). Five past medical history diagnoses (cancer, chronic obstructive pulmonary disease, dementia, myocardial infarction, and pulmonary embolism) were collected starting from 2012; therefore, these were missing in the 2009–2011 survey years. Information on whether the patient arrived on oxygen was not collected in the 2014 survey year.

#### Reason for visit

Reason for visit information, extracted using NLP, included up to three reasons for visit or cause of injury recorded by the providers for each patient in the ED triage notes. The survey methodology used in classifying patient reasons for visit has been described previously and is designed to approximate the patient’s own words [27]. The reason for visit classification system derived by the National Center for Health Statistics is a modular framework into which the reason for visit is broadly categorized as a type of complaint (e.g., symptoms, diseases, injury) and a methodology for systematically recording these complaints within a specific organ system or body area. The system then records the complaint in a pre-specified fashion according to an alphabetical index of complaints (for example, “eye pain” is changed to “pain, eye”) while maintaining the emphasis on the patient’s lay terminology rather than a clinician’s translation of the patient’s reason for visit.

#### Missing data

Missing values for age, sex, race, and ethnicity, approximately 0.1%, 0.9%, 16.8%, and 30.3% respectively, were imputed by the NHAMCS investigators in the dataset prior to public release. According to the NHAMCS, the investigators imputed age and sex using a hot deck based on 3-digit ICD-9-CM code for primary diagnosis, triage level, ED volume, and geographic region, while they imputed patient ethnicity using a model-based single, sequential regression method [26]. We imputed missing values for all other variables with the median of the corresponding variable before establishing the statistical models for this study; these variables include vital signs, mode of arrival, patient’s residence type, source of payment, episode of care, whether the visit was related to injury/poisoning, triage level, and pain scale (Table 1).

**Table 1:** Baseline characteristics of U.S. patients presenting to the ED, stratified by advanced imaging techniques, NHAMCS 2009–2014.

	Study Population	Any ADI	CT Only	US Only	MRI Only	Multiple ADI
	N = 139,150	N = 30,499(21.9%)	N = 23,327(16.8%)	N = 4,958(3.6%)	N = 587(0.4%)	N = 1,627(1.2%)
Age group*						
18–29 years	36,275(26.1)	6,293(20.6)	3,895(16.7)	2,015(40.6)	78(13.3)	305(18.7)
30–44 years	35,969(25.8)	7,028(23.0)	5,068(21.7)	1,415(28.5)	149(25.4)	396(24.3)
45–64 years	39,640(28.5)	8,888(29.1)	7,256(31.1)	926(18.7)	217(37.0)	489(30.1)
65–74 years	11,605(8.3)	3,208(10.5)	2,707(11.6)	250(5.0)	67(11.4)	184(11.3)
≥75 years	15,661(11.3)	5,082(16.7)	4,401(18.9)	352(7.1)	76(12.9)	253(15.6)
**Sex (Male %)**	60,103(43.2)	12,468(40.9)	10,519(45.1)	1,096(22.1)	245(41.7)	608(37.4)
**Ethnicity (Hispanic %)**	18,232(13.1)	4,103(13.5)	2,803(12.0)	985(19.9)	71(12.1)	244(15.0)
**Race**						
White	101,534(73.0)	23,398(76.7)	18,161(77.9)	3,534(71.3)	441(75.1)	1,262(77.6)
Black	31,765(22.8)	5,751(18.9)	4,201(18.0)	1,165(23.5)	110(18.7)	275(16.9)
Others	5,851(4.2)	1,350(4.4)	965(4.1)	259(5.2)	36(6.1)	90(5.5)
**Residence**						
Private residence	126,436(94.7)	27,505(93.9)	20,764(92.8)	4,688(97.7)	550(96.8)	1,503(96.0)
Nursing home	3,373(2.5)	1,109(3.8)	1,012(4.5)	53(1.1)	8(1.4)	36(2.3)
Homeless	1,514(1.1)	180(0.6)	153(0.7)	16(0.3)	4(0.7)	7(0.4)
Other	2,132(1.6)	510(1.7)	443(2.0)	41(0.9)	6(1.1)	20(1.3)
**Source of Payment**						
Private insurance	39,823(30.8)	9,382(32.6)	7,109(32.4)	1,520(32.3)	209(37.3)	544(35.9)
Medicare	31,758(24.6)	8,741(30.4)	7,416(33.8)	729(15.5)	154(27.5)	442(29.2)
Medicaid or CHIP	28,875(22.3)	5,385(18.7)	3,407(15.5)	1,578(33.6)	103(18.4)	297(19.6)
Uninsured	22,990(17.8)	4,107(14.3)	3,144(14.3)	709(15.1)	65(11.6)	189(12.5)
Other	5,829(4.5)	1,128(3.9)	888(4.0)	167(3.6)	29(5.2)	44(2.9)
**Day of Week**						
Sunday	19,142(13.8)	4,161(13.6)	3,276(14.0)	608(12.3)	65(11.1)	212(13.0)
Monday	21,982(15.8)	4,843(15.9)	3,661(15.7)	826(16.7)	95(16.2)	261(16.0)
Tuesday	20,299(14.6)	4,414(14.5)	3,316(14.2)	742(15.0)	87(14.8)	269(16.5)
Wednesday	19,985(14.4)	4,423(14.5)	3,387(14.5)	705(14.2)	104(17.7)	227(14.0)
Thursday	19,369(13.9)	4,290(14.1)	3,245(13.9)	717(14.5)	84(14.3)	244(15.0)
Friday	19,396(13.9)	4,273(14.0)	3,238(13.9)	727(14.7)	100(17.0)	208(12.8)
Saturday	18,977(13.6)	4,095(13.4)	3,204(13.7)	633(12.8)	52(8.9)	206(12.7)
**Arrival time**						
Morning	53,088(38.9)	11,840(39.5)	8,864(38.7)	2,025(41.2)	269(46.9)	682(42.6)
Afternoon	59,518(43.6)	12,996(43.3)	9,961(43.4)	2,117(43.1)	243(42.3)	675(42.2)
Evening	23,993(17.6)	5,176(17.2)	4,102(17.9)	768(15.6)	62(10.8)	244(15.2)
**Initial vital signs (mean±sd)**						
**Body Temperature**	36.73±0.52	36.72±0.53	36.71±0.54	36.75±0.45	36.74±0.48	36.74±0.56
**Heart rate**	85.94±17.61	85.36±17.80	85.15±18.09	86.21±16.33	84.31±16.78	86.28±18.20
**Respiratory rate**	18.55±4.54	18.63±4.33	18.68±4.43	18.47±4.07	18.31±4.99	18.65±3.39
**SBP**	135.98±23.32	137.92±25.10	139.26±25.46	131.09±21.62	138.99±24.25	139.01±26.61
**DBP**	79.28±14.55	79.21±15.14	79.59±15.38	77.14±13.83	79.98±14.23	79.73±15.29
**Pulse oximetry**	97.22±6.10	97.23±5.73	97.09±5.70	97.89±5.36	97.64±5.67	97.14±7.02
**Receiving oxygen on arrival**	5,739(5.8)	1,765(8.2)	1,477(9.0)	141(4.0)	21(5.5)	126(10.8)
**Pain Scale(mean±sd)**	5.16±3.67	5.57±3.59	5.51±3.63	5.85±3.25	5.79±3.74	5.50±3.78
** Follow-up visit to the ED (vs initial visit)**	9,260(7.3)	1,635(5.8)	1,045(4.9)	443(9.7)	56(10.4)	91(6.1)
**Visited last 72 hours**	6,041(5.0)	1,089(4.1)	779(3.8)	227(5.2)	38(7.2)	45(3.2)
**Arrived by Ambulance**	25,805(19.4)	8,531(29.1)	7,288(32.5)	650(13.7)	131(23.4)	462(29.5)
**Triage level**						
Immediate	1,606(1.3)	452(1.7)	364(1.8)	47(1.1)	4(0.8)	37(2.6)
Emergent	13,900(11.7)	4,148(15.6)	3,364(16.6)	452(10.4)	65(13.1)	267(19.0)
Urgent	57,246(48.1)	16,413(61.9)	12,352(61.0)	2,896(66.7)	280(56.6)	885(62.9)
Semi-urgent	38,247(32.1)	4,777(18.0)	3,646(18.0)	814(18.8)	126(25.5)	191(13.6)
Non-urgent	8,033(6.7)	717(2.7)	537(2.7)	132(3.0)	20(4.0)	28(2.0)
**Visit related to an injury, poisoning, or adverse effect of medical treatment**						
Yes	46,834(34.3)	9,047(30.2)	8,013(34.9)	560(11.5)	178(31.0)	296(18.6)
**Is injury/poisoning intentional**						
Not an injury visit	89,614(70.9)	20,911(73.5)	14,927(68.7)	4,290(92.5)	397(74.5)	1,297(83.9)
Yes, self-inflicted	1,508(1.2)	189(0.7)	174(0.8)	12(0.3)	1(0.2)	2(0.1)
Yes, assault	2,254(1.8)	842(3.0)	794(3.7)	30(0.6)	1(0.2)	17(1.1)
No, unintentional	33,026(26.1)	6,497(22.8)	5,827(26.8)	307(6.6)	134(25.1)	229(14.8)
**Past medical diagnoses**						
Myocardial infarction	2,534(4.2)	686(5.1)	571(5.5)	53(2.4)	14(5.0)	48(6.6)
Cancer	2,411(4.0)	722(5.3)	617(6.0)	50(2.3)	15(5.3)	40(5.5)
Cerebrovascular Disease	4,666(3.4)	1,850(6.1)	1,533(6.6)	104(2.1)	40(6.8)	173(10.6)
Congestive heart failure	5,166(3.7)	1,300(4.3)	1,061(4.5)	134(2.7)	22(3.7)	83(5.1)
Chronic obstructive pulmonary disease	3,126(5.1)	711(5.2)	599(5.8)	58(2.6)	17(6.0)	37(5.1)
Dementia	971(1.6)	405(3.0)	360(3.5)	22(1.0)	4(1.4)	19(2.6)
Diabetes	15,569(11.2)	3,947(12.9)	3,161(13.6)	456(9.2)	83(14.1)	247(15.2)
Pulmonary embolism	588(1.0)	184(1.4)	135(1.3)	29(1.3)	7(2.5)	13(1.8)
Condition requiring dialysis	1,502(1.1)	375(1.2)	292(1.3)	46(0.9)	10(1.7)	27(1.7)
HIV	940(0.7)	163(0.5)	131(0.6)	17(0.3)	4(0.7)	11(0.7)

*All variables were statistically significant (p<0.05)

Note: Missing values for *respiratory rate*, *systolic and diastolic blood pressure*, *arrival by ambulance*, *patient’s residence type*, *arrival time*, *and whether the visit is related to injury/poisoning* is lower than 5%. Missing values for *body temperature*, *heart rate*, *pulse oximetry source of payment*, *episode of care*, and *whether this injury/poisoning is intentional* is between 5% and 10%, and the missing values for whether patient been seen in this ED within the last 72 hours and triage level are between 10% and 15%. Missing values for pain scale is 22%. The analyses for cancer, chronic obstructive pulmonary disease, dementia, pulmonary embolism, myocardial infarction variables were calculated based on 2012–2014 data only (n = 60,906), since these comorbidities were not included in the dataset prior to 2012. The analysis for the variable *receiving oxygen on arrival* was done using 2009–2013 data only (n = 120,922), since this information was not collected in 2014.

### Statistical analyses

#### Topic modeling

NLP is a branch of computational linguistic techniques that extract and analyze information from unstructured and semi-structured text or speech data. Topic modeling is a commonly used technique for NLP, which can identify patterns hidden in the free text to evaluate an underlying theme or topic of the text [28]. The model based on the Latent Dirichlet Allocation (LDA) algorithm[29, 30] was used to break all the free text into different themes after preprocessing [31, 32].

The mathematical principles and algorithm of LDA have been described in prior research [28, 29]. Briefly, the free-text reasons for visit from each patient is a mixture of several topics composed of a set of words. For example, in a two-topic model, reasons for visit from patient 1 may contain 20% topic A (gastrointestinal problem) and 80% topic B (respiratory problems), while patient 2’s reason for visit could be 90% topic A and 10% topic B. The most common terms in the gastrointestinal topic might be “hematemesis” and “vomit”, while the respiratory problems may be composed of words including “breath”, “asthma”, and “shortness”. The LDA method can identify the mixture of topics, which describes each free-text reason for visit, while determining the mixture of words that associated with each topic. The correlation coefficient between each patient and each topic can be estimated. In this way, the free text reason for visit were transformed into a structured matrix of correlation coefficients between the patients and topics, which can be used for predicting the outcome. We employed the ldatatuning package in R for this analysis as previously described [32].

#### Regression modelling

Logistic regression models were used to measure the association between the outcome and the structured and unstructured predictors, and to predict the outcomes. To determine the predictive performance in identifying patients with advanced imaging use, we analyzed three models: (1) models with structured variables; (2) models with free-text data using NLP; (3) models with both structured and free-text variables. This was done for any ADI use, any CT use (including multiple cases), any U/S scan (including multiple cases), any MRI (including multiple cases), and multiple types of ADI use.

Ten-fold cross-validation was used to validate the performance of each model. The dataset was randomly divided into 10 sets; 9 of the 10 sets were used to train the models while the one remaining was used as the testing set. The area under the receiver-operating curve (ROC) was recorded for the testing set. The average ROC curve was derived by comparing the prediction values from all 10 cross-validation testing set. The probabilities of ADI use for each patient were calculated with this model. The best cutoff of the probabilities was determined by using the point on the ROC curve with the shortest distance to the upper left corner (where sensitivity = 1 and specificity = 1). The best cutoff of the probabilities for prediction and the corresponding sensitivity, specificity, and overall accuracy were recorded [33].

To evaluate the effect of the missing values on the models, specifically for the five comorbidities variables that were not part of the dataset prior to 2012 as well as one variable that was not collected in one survey year, we performed a sensitivity analysis using cases without missing values in any of the variables considered. Among a total of 139,150 cases, there were 14,009 (10.1%) cases without any missing values. Basic data organization was done in SAS 9.4. The text analyses were performed in R 3.3.2. The modeling of logistic regression was performed in Matlab R2016b.

## Results

### Characteristics of ED patients

Among 139,150 ED patient visits from December 2008 to December 2014, 21.9% of visits resulted in ADI use, including 16.8% who had CTs, 3.6% with U/S, 0.4% with MRIs, and 1.2% who had multiple types of ADI. The ADI use proportion increased in the older age groups. Females presented higher ADI use (22.8%) than males (20.7%). Hispanic patients had higher ADI use (22.5%) than non-Hispanics patients (21.8%) White had higher ADI use proportion (23%) than African American patients (18.1%). Patients from nursing home (32.9%) and private residence (21.8%) had higher proportion of ADI use than homeless patients (11.9%). Medicare patients (27.5%) and private insurance patients (23.6%) have higher ADI use proportions than Medicaid (18.6%) and of uninsured patients (17.9%). Patients who arrived by ambulance (33.1%) presented higher proportion of ADI use than patients those who did not (19.4%) (Table 1).

### Factors associated with ADI use

The adjusted odds ratio of ED visits resulting in different types of ADI use (vs. no ADI use) for each variable using multinomial logistic regression analyses are presented in Table 2 and S1 Table. Adjusted analyses showed male patients were 8% less likely to receive any ADI compared to female patients (OR: 0.92 95% CI 0.90–0.95). African American patients were 21% less likely to have ADI (OR: 0.79, 95% CI 0.76–0.82). Compared to those with private insurance, patients with Medicare were 24% less likely have ADI (OR: 0.76, 95% CI 0.73–0.79), while patients with Medicaid were 23% less likely (OR: 0.77, 95% CI 0.74–0.80) and uninsured patients were 22% less likely to have ADI (OR: 0.78, 95% CI 0.74–0.81). Unadjusted analyses are presented in S2 Table.

**Table 2:** Adjusted odds ratio of selected characteristics associated with the use of advanced diagnostic imaging studies during the emergency department visit (vs. no advanced imaging use), NHAMCS 2009–2014.

	Any ADI	CT Only	US Only	MRI Only	Multiple ADIs
**Age group**	** **				
18–29 years	Reference				
30–44 years	1.11(1.06–1.15)	1.28(1.22–1.34)	0.76(0.71–0.82)	1.84(1.39–2.43)	1.23(1.05–1.43)
45–64 years	1.25(1.20–1.30)	1.61(1.54–1.69)	0.51(0.46–0.55)	2.35(1.79–3.08)	1.25(1.07–1.46)
65–74 years	1.69(1.59–1.79)	2.22(2.08–2.37)	0.57(0.49–0.66)	3.00(2.06–4.35)	1.63(1.31–2.03)
≥75 years	1.97(1.86–2.08)	2.61(2.45–2.79)	0.65(0.56–0.75)	2.80(1.91–4.11)	1.64(1.32–2.04)
**Sex (male vs female)**	0.92(0.90–0.95)	1.06(1.02–1.09)	0.49(0.45–0.52)	0.95(0.80–1.12)	0.82(0.74–0.91)
**Ethnicity, Hispanic (vs Non-Hispanic)**	1.09(1.05–1.13)	0.99(0.95–1.04)	1.45(1.34–1.57)	0.98(0.76–1.26)	1.23(1.07–1.41)
**Race**	** **				
White	Reference				
African American	0.79(0.76–0.82)	0.77(0.74–0.80)	0.91(0.85–0.98)	0.80(0.64–0.99)	0.68(0.60–0.78)
Others	1.03(0.97–1.10)	0.96(0.89–1.04)	1.22(1.07–1.40)	1.44(1.02–2.03)	1.25(1.01–1.56)
**Residence**	** **				
Private residence	Reference				
Nursing home	0.90(0.83–0.98)	0.95(0.87–1.04)	0.67(0.50–0.90)	0.41(0.20–0.84)	0.49(0.34–0.69)
Homeless	0.54(0.46–0.64)	0.55(0.46–0.66)	0.44(0.27–0.73)	0.61(0.23–1.66)	0.49(0.23–1.03)
Other	1.03(0.93–1.15)	1.11(0.99–1.25)	0.73(0.53–1.00)	0.67(0.30–1.51)	0.79(0.51–1.24)
**Arrived by Ambulance**	1.87(1.80–1.93)	2.01(1.93–2.09)	1.13(1.03–1.24)	1.69(1.36–2.09)	2.00(1.77–2.27)
**Source of payment**	** **				
Private Insurance	Reference				
Medicare	0.76(0.73–0.79)	0.78(0.74–0.81)	0.70(0.64–0.77)	0.62(0.49–0.79)	0.69(0.60–0.80)
Medicaid or CHIP	0.77(0.74–0.80)	0.68(0.65–0.71)	1.07(0.99–1.15)	0.70(0.55–0.89)	0.72(0.62–0.84)
Uninsured	0.78(0.74–0.81)	0.80(0.76–0.84)	0.74(0.67–0.81)	0.60(0.45–0.80)	0.65(0.55–0.77)
Other	0.83(0.77–0.89)	0.82(0.76–0.89)	0.95(0.81–1.13)	0.98(0.66–1.45)	0.65(0.47–0.88)
**Receiving oxygen on arrival**	0.93(0.88–1.00)	0.93(0.87–1.00)	0.87(0.73–1.05)	0.72(0.45–1.14)	1.20(0.97–1.47)
**Follow up visit to the ED vs. initial visit**	0.88(0.82–0.93)	0.73(0.68–0.79)	1.43(1.28–1.61)	1.36(0.99–1.86)	0.97(0.77–1.21)
**Visited ED in last 72 hours**	0.85(0.79–0.92)	0.87(0.80–0.95)	0.79(0.67–0.92)	1.24(0.85–1.80)	0.62(0.45–0.85)
**Triage level**	** **				
Non-urgent	Reference				
Immediate	3.21(2.80–3.68)	3.15(2.70–3.67)	2.83(2.01–4.00)	1.21(0.41–3.55)	6.30(3.82–10.41)
Emergent	3.48(3.19–3.80)	3.52(3.19–3.89)	2.98(2.44–3.64)	2.11(1.27–3.51)	5.15(3.47–7.64)
Urgent	3.11(2.87–3.37)	3.05(2.78–3.34)	3.28(2.75–3.91)	2.13(1.35–3.36)	4.05(2.78–5.91)
Semi-urgent	1.34(1.23–1.46)	1.33(1.21–1.46)	1.39(1.15–1.67)	1.32(0.82–2.12)	1.41(0.95–2.10)
**Visit related to an injury, poisoning, or adverse effect of medical treatment**					
Yes (vs No)	0.61(0.57–0.65)	0.67(0.63–0.71)	0.48(0.42–0.56)	0.86(0.62–1.18)	0.32(0.24–0.43)
**Is the injury/poisoning intentional**					
Not an injury/poisoning visit	Reference				
Yes, self-inflicted	0.74(0.63–0.87)	0.87(0.73–1.04)	0.35(0.19–0.63)	0.18(0.02–1.32)	0.23(0.06–0.96)
Yes, assault	3.85(3.46–4.28)	4.84(4.33–5.42)	0.80(0.54–1.19)	0.20(0.03–1.48)	2.66(1.52–4.66)
No, unintentional	1.36(1.28–1.45)	1.53(1.43–1.64)	0.47(0.39–0.56)	1.01(0.71–1.43)	1.57(1.15–2.15)
**Pain Scale**	** **				
0–2	Reference				
3–6	1.40(1.35–1.45)	1.38(1.33–1.44)	1.54(1.41–1.68)	1.31(1.05–1.65)	1.21(1.06–1.39)
7–10	1.86(1.79–1.93)	1.89(1.81–1.97)	1.76(1.61–1.92)	1.91(1.51–2.41)	1.86(1.62–2.13)
**Cerebrovascular Disease**	1.74(1.63–1.85)	1.71(1.60–1.84)	1.08(0.88–1.33)	2.15(1.53–3.02)	3.20(2.68–3.81)
**Congestive heart failure**	0.74(0.69–0.80)	0.72(0.67–0.78)	0.98(0.81–1.18)	0.75(0.48–1.17)	0.78(0.62–0.99)
**Diabetes**	0.96(0.92–1.00)	0.95(0.91–1.00)	1.01(0.91–1.12)	1.03(0.81–1.32)	1.04(0.90–1.21)
**Condition requiring dialysis**	0.92(0.82–1.05)	0.88(0.77–1.01)	1.11(0.82–1.51)	1.38(0.73–2.62)	1.07(0.72–1.59)
**HIV**	0.82(0.69–0.98)	0.88(0.72–1.06)	0.56(0.34–0.90)	0.96(0.36–2.60)	1.03(0.57–1.89)
**Cancer**	1.13(1.03–1.24)	1.18(1.07–1.31)	0.79(0.59–1.06)	1.04(0.61–1.77)	1.09(0.78–1.52)
**Chronic obstructive pulmonary disease**	0.76(0.69–0.83)	0.78(0.71–0.87)	0.63(0.48–0.83)	0.86(0.52–1.44)	0.64(0.45–0.90)
**Dementia**	1.56(1.35–1.80)	1.58(1.36–1.83)	1.42(0.90–2.24)	1.00(0.36–2.79)	1.44(0.87–2.37)
**Pulmonary embolism**	1.21(1.01–1.46)	1.08(0.88–1.33)	1.89(1.28–2.79)	2.30(1.06–4.98)	1.40(0.79–2.47)
**Myocardial infarction**	0.88(0.80–0.97)	0.87(0.79–0.97)	0.88(0.66–1.18)	0.82(0.47–1.45)	0.98(0.71–1.35)

Note: The odds ratios for any ADI use were estimated with binary logistic regression. The odds ratios for other ADI uses were estimated with multivariable logistic regression, and were adjusted for all other variables listed in the Table 1. The odds ratios for *cancer*, *chronic obstructive pulmonary disease*, *dementia*, *pulmonary embolism*, *myocardial infarction* were calculated using 2012–2014 data only, since these comorbidities were not collected prior to 2012. The odds ratio of *receiving oxygen on arrival* were based 2009–2013 data only, since this variable was not collected in 2014.

Age, triage level, arrival mode, place of residence, and certain comorbidities were also predictive of the eventual use of ADI. For example, the odds of ADI use increased progressively with increasing age; compared to patients in the age 18–29 group, the adjusted odds of ADI use was 1.97 times higher for patients ≥ 75 years old (95% CI 1.86–2.08), 1.69 times higher for patients in the 65–74 age group (95% CI 1.59–1.79), 1.25 times higher for patients in the 45–64 age group (95% CI 1.20–1.30), and 1.11 times higher for patients in the 30–44 age group (95% CI 1.06–1.15) (Table 2). Those who arrived via ambulance were 1.87 times more likely to receive ADI than those who did not (95% CI 1.80–1.93). Compared to those who lived in a private residence, nursing home patients were 10% less likely (OR: 0.90, 95% CI 0.83–0.98), while those who were homeless were 46% less likely to have any ADI use (OR: 0.54 95% CI 0.46–0.64). Comorbidities had varying likelihood of any ADI use; patients with history of cerebrovascular disease were 1.74 times more likely (95% CI 1.63–1.85) and those with dementia were 1.56 times more likely (95% CI 1.35–1.80) than those who did not have respective comorbidities to have any ADI use (Table 2). Increasing pain scale was associated progressively with an increased likelihood of any ADI use, as well as of CT use (S1 Fig). The trends in the likelihood of CT use only and multiple types of ADI use (vs. no ADI use) closely mirrored that of any ADI use with race, age, triage level, mode arrival, place of residence, source of payment, and certain comorbidities (cerebrovascular disease and dementia) being most predictive based on the odds ratios (Table 2).

### Text variables extracted using topic modelling to predict ADI use

The top 10 terms in each topic for the first 20 topics are presented in S3 Table. Although these topics cannot all be generalized into terms that are clinically meaningful, words that have been grouped into topics may indicate a theme. For example, the first topic shows a theme related to the extremities, the second gastrointestinal problems, the third respiratory problems, and the fourth trauma. The first 20 topics all show significant odds in all types of ADI use; for example, “topic 12” has an odds ratio of 0.07 (95% CI 0.04–0.11) for predicting any ADI use.

### Predictive performance of multivariable logistic regression models

**Fig 1:**
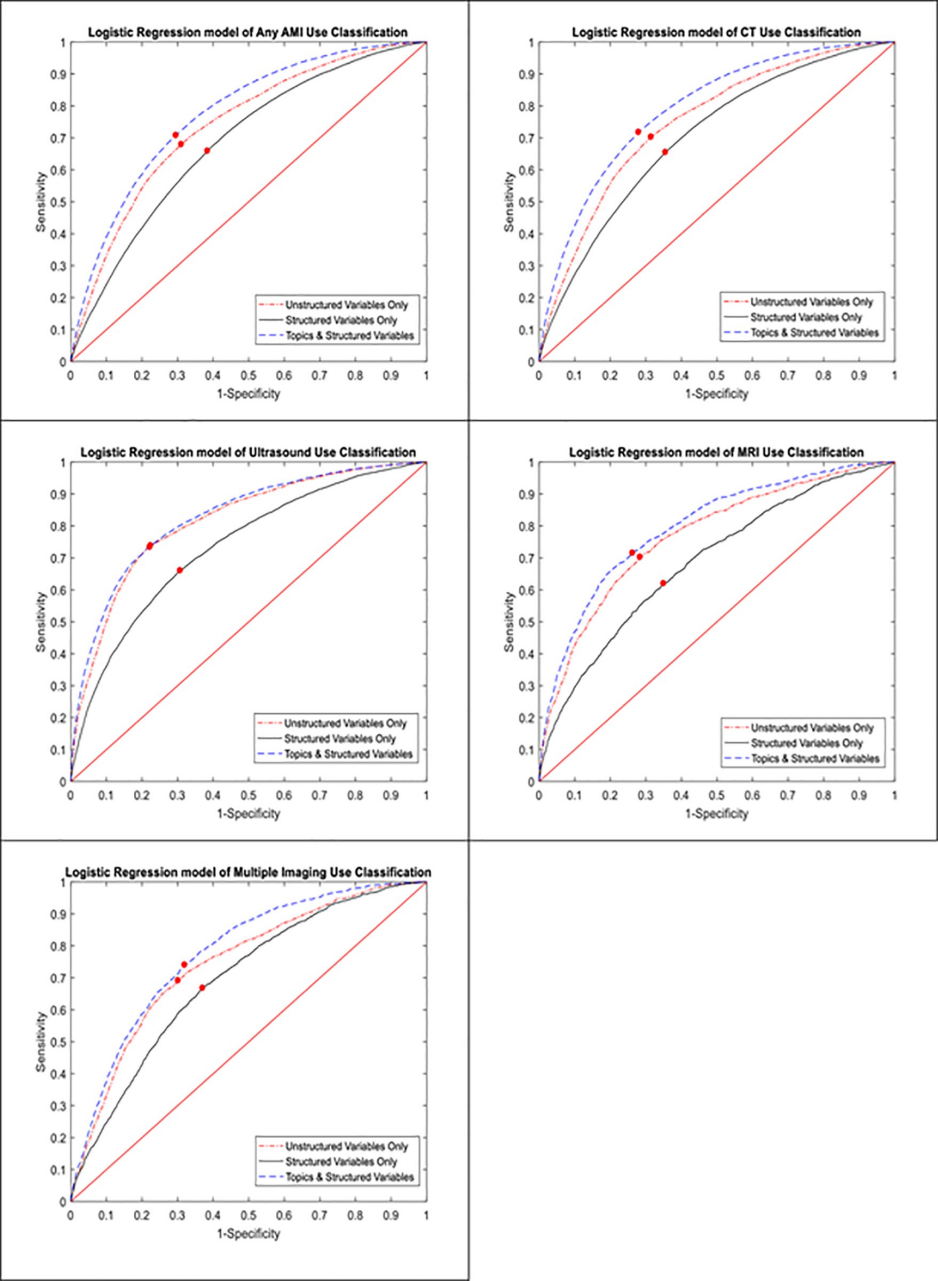
Receiver operating curves for the logistic regression models for different types of advanced diagnostic imaging use (Any ADI, CT, MRI, US, Multiple ADI). The red point on the curve minimized the Euclidian distance between the ROC curve and the upper left coordinate which defined the best cutoff for this study.

Applying the three logistic regression models (model 1: structured variables only, model 2: text-based reason for visit variables only, and model 3: both text-based and structured variables), we found that the predictive accuracy for ADI use was higher for models with text-based reason for visit variables only compared to models with structured variables only. The predictive accuracy was the highest when both text-based reason for visit and structured variables were included (Table 3 and Fig 1). For models that included both unstructured and unstructured variables, the AUC was 0.78 (0.77–0.78) for any ADI use, 0.79 (0.79–0.79) for CT use, 0.83 (0.82–0.84) for U/S use, 0.80 (0.79–0.80) for MRI use, and 0.78 (0.77–0.79) for multiple ADI use.

**Table 3:** Predictive performance of logistic regression models with 10-fold classification in identifying patients with various advanced imaging use during emergency department triage, NHAMCS 2009–2014.

	Probability cut-off	Sensitivity	Specificity	Accuracy	AUC(95% CI)
**Any ADI use**					
Unstructured variables	0.22	0.68	0.69	0.69	0.74(0.73–0.75)
Structured variables	0.22	0.66	0.62	0.63	0.69(0.68–0.69)
Unstructured + Structured variables	0.22	0.71	0.70	0.71	0.78(0.77–0.78)
**CT Use**					
Unstructured variables	0.17	0.70	0.69	0.69	0.75(0.75–0.75)
Structured variables	0.18	0.66	0.65	0.65	0.70(0.70–0.71)
Unstructured + Structured variables	0.19	0.72	0.72	0.72	0.79(0.79–0.79)
**Ultrasound Use**					
Unstructured variables	0.04	0.73	0.78	0.78	0.82(0.81–0.82)
Structured variables	0.05	0.66	0.69	0.69	0.74(0.73–0.74)
Unstructured + Structured variables	0.04	0.74	0.78	0.77	0.83(0.82–0.84)
**MRI Use**					
Unstructured variables	0.01	0.70	0.72	0.72	0.77(0.76–0.78)
Structured variables	0.01	0.62	0.65	0.65	0.69(0.68–0.70)
Unstructured + Structured variables	0.01	0.72	0.74	0.74	0.80(0.79–0.80)
**Multiple ADI Use**					
Unstructured variables	0.01	0.69	0.70	0.70	0.75(0.74–0.75)
Structured variables	0.01	0.67	0.63	0.63	0.70(0.69–0.71)
Unstructured + Structured variables	0.01	0.74	0.68	0.68	0.78(0.77–0.79)

Note: The best cutoff of the probabilities was determined by using the point on the ROC curve with the shortest distance to the upper left corner (where sensitivity = 1 and specificity = 1). The unstructured variables refer to the correlation coefficients between the patient and topics from the free-text reasons for visit.

Estimated coefficients and standardized coefficients of the structured variables from logistic regression between the outcome of ADI use and the predictors were presented as a modeling example (S4 Table), which can be used for perspective study. Standardized coefficients can be compared to present which variable have a greater effect on the ADI use prediction. The item “whether the injury/poisoning intentional” and the immediate triage level presented highest standardized coefficients among the structured variables.

We performed a sensitivity analysis for missing values. Among the 139,150 records, there were 14,009 records without any missing values of which 3,409 (24.4%) resulted in ADI use, including 2,560 (18.3%) CTs, 613 (4.38%) USs, 65 (0.46%) MRIs, and 171 (1.22%) multiple AMIs. With 100 topic models from the unstructured data included, the AUC was 0.76 (95% CI 0.75–0.77). With the structured variables included only, the AUC was 0.70 (95% CI 0.68–0.72). With both the structured variables and 80 topics from unstructured data included, the AUC reached 0.79 (95% CI 0.78–0.81).

## Discussion

Improving ED efficiency may help address the continued problem and negative consequences of ED crowding in the U.S. [2, 6, 34]. One previously unexplored solution to address this problem may be to identify patients more likely to eventually obtain ADI earlier in their ED encounter. Our study applied predictive analytics and natural language processing modeling techniques with six years of nationally representative survey data to create a model to predict ADI use during the ED triage process.

One of the novel aspects of this study was the use of not only structured variables (examples: age, race, residence type), but also text-based information (reason for visit and cause of injury) via natural language processing. Specifically, we chose LDA topic modelling, which balances predictive performance and ease of information interpretation by grouping words into topics [28]. With the inclusion of reason for visit information in the model, the AUC ranged from 0.78 to 0.83 for all outcomes. When choosing the best probability cut-off given in the study (p = 0.05) as the threshold, the best overall accuracy of this model for ultrasound use, for example, reached 78% (with sensitivity of 0.73 and specificity of 0.78), which means that with the model given in the study, physicians can predict with an accuracy of 73% whether a patient will eventually receive ultrasound during their ED stay, and offers a 78% discriminatory accuracy for those who will not receive ADI.

During our exploration and model development, we observed surprising and substantial racial and socioeconomic disparities in the use of ADI in this sample. Similar to previous studies, African Americans were less likely to have ADI compared to white patients [35, 36]. There are several potential explanations for these differences. For example, some evidence suggests that injury severity varies by race, thus warranting differential use of ADI [35, 37]. In addition, the extent of overcrowding in an ED has been shown to affect the thoroughness of patients’ evaluation, which disproportionately affects hospitals that serve higher number of African Americans [37, 38]. Other potential explanations for racial differences in ADI use include provider implicit bias [35], and/or potential overuse of ADI in white patients rather than underuse by African American patients [39].

Patients with Medicaid and uninsured patients were also less likely to receive ADI compared to patients with private insurance [40]. Compared to patients that live in private residences, patients from nursing homes and patients who were homeless had decreased likelihood of any ADI use. Reasons for these disparities should be further explored in future research to determine the appropriateness of including or excluding these variables in prediction models [37] based on the clinical context, such as the one proposed in this study. This will be important to determine whether such prediction models can serve as a more objective tool to predict whether a patient will need ADI by excluding factors that may be influenced by clinician bias, for example. It may also be of value to explore the relationship between measurements of disparities, such as the role that insurance type plays in the racial differences we observed in ADI, or the influence of the ED specific characteristics such as urbanicity, teaching hospital designation, or safety net designation on ADI utilization. Further studies are needed to determine the effect of predictive clinical decision support algorithms such as the one constructed for this study, on the clinical use of ADI in settings where it can potentially be deployed to reduce racial and socioeconomic disparities in ADI use.

We also found that age, triage level, arrival mode, place of residence, and certain comorbidities were predictive of the eventual use of ADI during ED visit. As expected, patients with emergent and immediate triage levels had the highest likelihood of ADI use. These patients are often immediately placed in an ED room for workup shortly after arrival and early identification of their need for ADI would likely have less of an impact on ED LOS, and the decision tree that can lead to ADI in these patients often bypasses the traditional triage processes. However, patients who were triaged as urgent (typically triage level 3) or semi-urgent (typical triage level 4) also had increased odds of ADI use. These patients typically spend a longer portion of their LOS in the waiting area prior to being placed in a room—after which a provider typically makes the decision to pursue ADI. Urgent and semi-urgent patients comprise the majority of all ED patients (80.2% in this sample) and stand to benefit the most from this form of predictive modelling as they utilize the majority of ED ADI (79.8% of ADI in this sample).

When the use of each type of ADI (CT, MRI, U/S) was analyzed, we found that the general trends closely mirrored that of any ADI use except for U/S. One explanation for this difference is the increasing number of ultrasounds performed unofficially as a point of care test at bedside or in a fashion that was not captured in the dataset. For example, the Focused Assessment with Sonography in Trauma (FAST) ultrasound [41] is often not captured, which would result in under-reporting in the current dataset. Despite having been shown in prior research to be a poor predictor of health outcomes[42, 43], we found that patients indicating higher levels of pain on the traditional ten-point pain scale had increased odds of receiving ADI (Table 2, S1 Fig). This may reflect the fact that physicians tend to do more for patients who complain of severe pain[44].

The triage process in the ED represents the earliest in-person point of contact between a patient and a medical provider, often a nurse, after arriving in the ED. This is an extremely important encounter, but one that is often quite brief. A decision support system built on models such as the ones proposed in this study may be valuable to triage personnel, charge nurses, hospital leaders, hospital flow coordinators, and ED physicians. Further research will be needed to test the effect of using such a system in the clinical setting. Because these models were derived from nationally representative survey data, the clinical use of this type of modelling strategy may benefit from location-specific data and additional calibration of models for specific regions of the county or patient populations.

The present study has several limitations. First, missing values in the datasets affect the predictive performance of the models; sensitivity analyses were performed to limit this potential source of bias. Second, the interpretation of the topic models used for natural language processing in extracting data from unstructured information is not always straightforward as these topics are computer generated and take into account multiple layers of variable interactions. Third, the specific test ordered (example: CT of head) was not available in the dataset for analysis; however, this may be important to explore in future studies. Fourth, the dataset does not provide the results of ADI studies, the medical indication for those studies, who ordered the study, or the time of ordering the study. Therefore, we lack the temporal information to know if an ADI study was ordered for a patient immediately upon arrival by triage personnel or if was ordered later by a different provider who was privy to additional clinical information such as lab results or changes in clinical status. Additionally, we used the ED physicians’ decision to pursue ADI as the gold standard to establish the outcome for the predictive model—not whether the imaging yielded or ruled out a diagnosis. As a result, the appropriateness of these decisions cannot be assessed. To determine the utility of these models in supporting physicians’ ED triage decisions, these predictive models should be developed and prospectively validated in the clinical setting. Because these predictive models were designed on national data and focused on a specific time point (triage) during an ED encounter, it is not possible to account for the impact of serial interactions in the ED that lead toward or away from ADI. Further studies are needed to assess the impact of using this type of predictive model on the triage behaviors, ordering patterns, clinical pathways, overall imaging utilization, and ED flow.

## Conclusion

This investigation used six years of nationally representative ED data to construct statistical models to predict the eventual use of advanced diagnostic imaging—using only the information that would be available at the time of ED triage: vital signs, general medical information, and the patient’s stated reason for visit. The overall discriminatory accuracy of these models supports prospective testing for use as an adjunct clinical decision support tool.

## Supporting information

S1 Fig **Adjusted Odds of receiving CT (left) and Ultrasound (right) by pain scale score**.
(TIF)

S1 Table Adjusted odds ratio of selected characteristics associated with the use of advanced diagnostic imaging studies during the emergency department visit (vs. no advanced imaging use), NHAMCS 2009–2014. (DOCX)

S2 Table Crude odds ratio of characteristics associated with the use of various types of advanced imaging studies during an emergency department visit (vs. no advanced imaging use), NHAMCS 2009–2014. (DOCX)

S3 Table Most frequent 10 words in each topic model for Topics 1–20. (DOCX)

S4 Table Parameter estimation between the outcome of ADI use and structured imaging during the emergency department visit, NHAMCS 2009–2014. (DOCX)
