# Supplementary figures and images for: Advanced diagnostic imaging utilization during emergency department visits in the United States: A predictive modeling study for emergency department triage
Source: PLoS One. 2019 Apr 9;14(4):e0214905. doi: 10.1371/journal.pone.0214905 (PMC6456195; doi:10.1371/journal.pone.0214905)

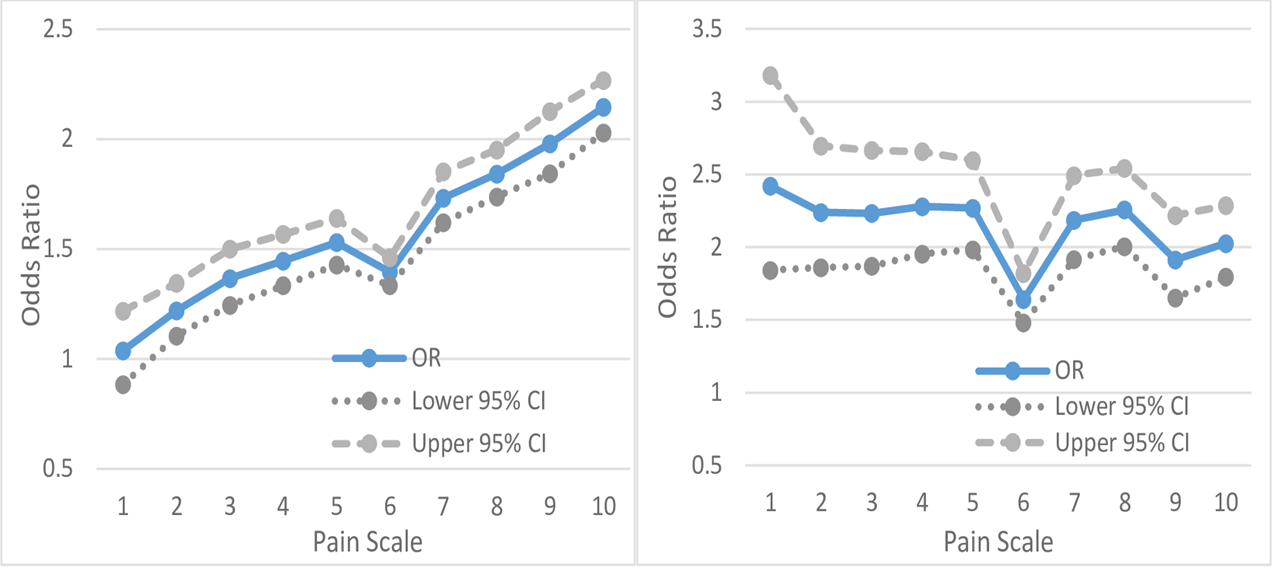

Supplement: S1 Fig — Adjusted Odds of receiving CT (left) and Ultrasound (right) by pain scale score. (TIF) [file pone.0214905.s001.tif]
