# Supplementary material for: Advanced diagnostic imaging utilization during emergency department visits in the United States: A predictive modeling study for emergency department triage
Source: PLoS One. 2019 Apr 9;14(4):e0214905. doi: 10.1371/journal.pone.0214905 (PMC6456195; doi:10.1371/journal.pone.0214905)
Supplement: S1 Table — (DOCX) [file pone.0214905.s002.docx]

**S1 Table.** Adjusted odds ratio of selected characteristics associated with the use of advanced diagnostic imaging studies during the emergency department visit (vs. no advanced imaging use), NHAMCS 2009-2014

|  | **Any ADI** | **CT Only** | **US Only** | **MRI Only** | **Multiple ADIs** |
| --- | --- | --- | --- | --- | --- |
| **Day of Week** |  |  |  |  |  |
| Sunday | Reference |  |  |  |  |
| Monday | 1.02(0.97-1.07) | 0.99(0.94-1.04) | 1.16(1.04-1.30) | 1.25(0.91-1.71) | 1.05(0.87-1.26) |
| Tuesday | 1.01(0.96-1.06) | 0.97(0.92-1.03) | 1.12(1.00-1.25) | 1.25(0.91-1.73) | 1.19(0.99-1.42) |
| Wednesday | 1.02(0.97-1.07) | 1.00(0.94-1.05) | 1.09(0.97-1.21) | 1.52(1.11-2.07) | 1.00(0.82-1.20) |
| Thursday | 1.02(0.97-1.07) | 0.99(0.93-1.04) | 1.14(1.02-1.28) | 1.27(0.91-1.75) | 1.11(0.92-1.34) |
| Friday | 1.02(0.97-1.07) | 0.98(0.93-1.04) | 1.19(1.06-1.33) | 1.50(1.09-2.05) | 0.95(0.79-1.16) |
| Saturday | 0.98(0.94-1.04) | 0.97(0.92-1.03) | 1.07(0.96-1.21) | 0.80(0.55-1.15) | 0.97(0.80-1.18) |
| **Arrival time** |  |  |  |  |  |
| Morning | Reference |  |  |  |  |
| Afternoon | 0.98(0.96-1.01) | 1.02(0.99-1.05) | 0.89(0.84-0.95) | 0.85(0.71-1.00) | 0.91(0.81-1.01) |
| Evening | 0.93(0.89-0.96) | 0.99(0.95-1.03) | 0.78(0.71-0.85) | 0.55(0.42-0.72) | 0.76(0.65-0.88) |
| **Initial vital sign** |  |  |  |  |  |
| **Temperature** |  |  |  |  |  |
| 36 C-38 C | Reference |  |  |  |  |
| <36 C | 1.03(0.97-1.09) | 1.08(1.01-1.15) | 0.81(0.69-0.94) | 0.76(0.51-1.14) | 1.13(0.92-1.39) |
| >38 C | 0.87(0.79-0.96) | 0.93(0.84-1.04) | 0.59(0.46-0.76) | 0.64(0.30-1.36) | 1.07(0.77-1.47) |
| Pulse oximetry % | 0.76(0.72-0.80) | 0.73(0.70-0.77) | 0.85(0.76-0.94) | 0.88(0.66-1.17) | 0.71(0.59-0.85) |
| <=90 vs >90 (normal) |  |  |  |  |  |
| **Heart rate** |  |  |  |  |  |
| 60-100 | Reference |  |  |  |  |
| < 60 | 0.98(0.93-1.04) | 1.04(0.98-1.10) | 0.75(0.66-0.86) | 0.94(0.68-1.30) | 1.00(0.82-1.21) |
| > 100 | 0.99(0.95-1.03) | 1.02(0.98-1.07) | 0.85(0.78-0.92) | 0.88(0.69-1.12) | 1.06(0.93-1.21) |
| **Respiratory rate** | 1.00(0.99-1.00) | 1.00(0.99-1.00) | 1.00(0.99-1.00) | 0.98(0.96-1.01) | 0.99(0.98-1.00) |
| **DBP** |  |  |  |  |  |
| 60-80 | Reference |  |  |  |  |
| <60 | 1.01(0.95-1.06) | 1.02(0.96-1.08) | 1.03(0.92-1.15) | 0.68(0.47-1.00) | 0.97(0.80-1.18) |
| >80 | 1.00(0.97-1.03) | 1.03(1.00-1.07) | 0.84(0.79-0.90) | 0.88(0.73-1.05) | 1.05(0.94-1.17) |
| **SBP** |  |  |  |  |  |
| 80-120 | Reference |  |  |  |  |
| < 80 | 0.75(0.68-0.84) | 0.77(0.68-0.86) | 0.69(0.55-0.88) | 1.19(0.61-2.30) | 0.96(0.66-1.41) |
| > 120 | 1.01(0.98-1.05) | 1.06(1.02-1.11) | 0.93(0.86-0.99) | 1.07(0.86-1.33) | 0.97(0.85-1.11) |

Note: This is a continuation of Table 2. The odds ratios for any ADI use were estimated with binary logistic regression. The odds ratios for other ADI uses were estimated with multivariable logistic regression, and were adjusted for all other variables listed in the Table 1.
