# Supplementary material for: Advanced diagnostic imaging utilization during emergency department visits in the United States: A predictive modeling study for emergency department triage
Source: PLoS One. 2019 Apr 9;14(4):e0214905. doi: 10.1371/journal.pone.0214905 (PMC6456195; doi:10.1371/journal.pone.0214905)
Supplement: S2 Table — (DOCX) [file pone.0214905.s003.docx]

**S2 Table**. Crude odds ratio of characteristics associated with the use of various types of advanced imaging studies during an emergency department visit (vs. no advanced imaging use), NHAMCS 2009-2014

|  | Any ADI | CT Only | US Only | MRI Only | Multiple |
| --- | --- | --- | --- | --- | --- |
| **Age group** |  |  |  |  |  |
| 18-29 years | Reference |  |  |  |  |
| 30-44 years | 1.16(1.11-1.20) | 1.35(1.29-1.41) | 0.73(0.68-0.78) | 1.98(1.50-2.60) | 1.35(1.16-1.56) |
| 45-64 years | 1.37(1.33-1.43) | 1.82(1.74-1.89) | 0.45(0.41-0.49) | 2.71(2.09-3.51) | 1.56(1.35-1.81) |
| 65-74 years | 1.82(1.73-1.91) | 2.48(2.35-2.62) | 0.44(0.39-0.51) | 3.07(2.21-4.25) | 2.15(1.79-2.59) |
| ≥75 years | 2.29(2.19-2.39) | 3.20(3.05-3.36) | 0.50(0.44-0.56) | 2.76(2.01-3.79) | 2.35(1.99-2.78) |
| **Sex (male vs female)** | 0.89(0.86-0.91) | 1.05(1.02-1.08) | 0.36(0.34-0.39) | 0.92(0.78-1.08) | 0.76(0.69-0.85) |
| **Ethnicity (Hispanic vs Non-Hispanic)** | 1.04(1.00-1.08) | 0.91(0.88-0.95) | 1.66(1.54-1.78) | 0.92(0.72-1.18) | 1.18(1.03-1.35) |
| **Race** |  |  |  |  |  |
| White | Reference |  |  |  |  |
| African American | 0.74(0.72-0.76) | 0.69(0.67-0.72) | 0.99(0.93-1.06) | 0.75(0.61-0.92) | 0.65(0.57-0.75) |
| Others | 1.00(0.94-1.07) | 0.92(0.86-0.99) | 1.27(1.12-1.45) | 1.42(1.01-2.00) | 1.24(1.00-1.54) |
| **Residence** |  |  |  |  |  |
| Private residence | Reference |  |  |  |  |
| Nursing home | 1.76(1.64-1.90) | 2.13(1.97-2.30) | 0.49(0.38-0.65) | 0.64(0.32-1.28) | 1.05(0.75-1.46) |
| Homeless | 0.49(0.42-0.57) | 0.55(0.46-0.65) | 0.25(0.16-0.42) | 0.54(0.20-1.44) | 0.35(0.16-0.73) |
| Other | 1.13(1.02-1.25) | 1.30(1.17-1.45) | 0.53(0.39-0.73) | 0.67(0.30-1.49) | 0.81(0.52-1.27) |
| **Arrived by Ambulance** | 2.06(1.99-2.12) | 2.41(2.33-2.49) | 0.79(0.73-0.86) | 1.53(1.26-1.86) | 2.10(1.88-2.34) |
| **Source of payment** |  |  |  |  |  |
| Private Insurance | Reference |  |  |  |  |
| Medicare | 1.23(1.19-1.27) | 1.38(1.33-1.43) | 0.63(0.58-0.69) | 0.97(0.79-1.20) | 1.07(0.95-1.22) |
| Medicaid or CHIP | 0.74(0.72-0.77) | 0.62(0.59-0.65) | 1.35(1.25-1.45) | 0.64(0.50-0.81) | 0.71(0.61-0.82) |
| Uninsured | 0.71(0.68-0.74) | 0.71(0.68-0.75) | 0.75(0.69-0.82) | 0.50(0.38-0.66) | 0.56(0.47-0.66) |
| Other | 0.78(0.73-0.83) | 0.81(0.75-0.87) | 0.71(0.60-0.84) | 0.90(0.61-1.33) | 0.52(0.38-0.71) |
| **Day of Week** |  |  |  |  |  |
| Sunday | Reference |  |  |  |  |
| Monday | 1.02(0.97-1.07) | 0.98(0.93-1.03) | 1.19(1.07-1.32) | 1.28(0.93-1.75) | 1.08(0.90-1.29) |
| Tuesday | 1.00(0.95-1.05) | 0.95(0.91-1.01) | 1.15(1.03-1.28) | 1.26(0.91-1.74) | 1.20(1.00-1.43) |
| Wednesday | 1.02(0.98-1.07) | 1.00(0.94-1.05) | 1.12(1.00-1.25) | 1.54(1.13-2.10) | 1.03(0.85-1.24) |
| Thursday | 1.02(0.98-1.08) | 0.98(0.93-1.04) | 1.17(1.05-1.31) | 1.28(0.93-1.78) | 1.14(0.95-1.38) |
| Friday | 1.02(0.97-1.07) | 0.98(0.93-1.03) | 1.18(1.06-1.32) | 1.52(1.11-2.08) | 0.97(0.80-1.18) |
| Saturday | 0.99(0.94-1.04) | 0.98(0.93-1.04) | 1.05(0.94-1.17) | 0.81(0.56-1.16) | 0.98(0.81-1.19) |
| **Arrival time** |  |  |  |  |  |
| Morning | Reference |  |  |  |  |
| Afternoon | 0.97(0.95-1.00) | 1.00(0.97-1.03) | 0.93(0.87-0.99) | 0.80(0.67-0.95) | 0.88(0.79-0.98) |
| Evening | 0.96(0.92-0.99) | 1.01(0.97-1.05) | 0.82(0.76-0.90) | 0.51(0.39-0.66) | 0.77(0.67-0.89) |
| **Initial vital sign** |  |  |  |  |  |
| **Temperature** |  |  |  |  |  |
| 36 C-38 C | Reference |  |  |  |  |
| <36 C | 1.09(1.03-1.15) | 1.18(1.11-1.25) | 0.70(0.60-0.81) | 0.79(0.53-1.18) | 1.20(0.98-1.48) |
| >38 C | 1.04(0.94-1.14) | 1.09(0.99-1.21) | 0.71(0.56-0.91) | 0.64(0.30-1.34) | 1.41(1.03-1.94) |
| Pulse oximetry % | 0.70(0.67-0.73) | 0.68(0.65-0.71) | 0.78(0.71-0.85) | 0.78(0.60-1.01) | 0.66(0.56-0.78) |
| <=90 vs >90 (normal) |  |  |  |  |  |
| **Heart rate** |  |  |  |  |  |
| 60-100 | Reference |  |  |  |  |
| <60 | 0.88(0.84-0.92) | 0.93(0.88-0.97) | 0.68(0.60-0.76) | 0.86(0.65-1.15) | 0.90(0.76-1.08) |
| >100 | 1.02(0.99-1.06) | 1.04(1.00-1.08) | 0.93(0.86-1.00) | 0.81(0.64-1.02) | 1.14(1.01-1.29) |
| **Respiratory rate** | 1.01(1.00-1.01) | 1.01(1.00-1.01) | 1.00(0.99-1.00) | 0.99(0.96-1.01) | 1.01(1.00-1.02) |
| **DBP** |  |  |  |  |  |
| 60-80 | Reference |  |  |  |  |
| <60 | 1.01(0.97-1.05) | 1.05(1.00-1.10) | 0.87(0.79-0.95) | 0.72(0.53-0.99) | 1.05(0.89-1.24) |
| >80 | 1.00(0.97-1.03) | 1.07(1.04-1.11) | 0.72(0.67-0.76) | 0.95(0.80-1.12) | 1.05(0.95-1.16) |
| **SBP** |  |  |  |  |  |
| 80-120 | Reference |  |  |  |  |
| <80 | 0.67(0.61-0.72) | 0.72(0.65-0.79) | 0.47(0.39-0.57) | 0.80(0.47-1.34) | 0.73(0.54-1.00) |
| >120 | 1.06(1.03-1.10) | 1.19(1.15-1.23) | 0.67(0.63-0.71) | 1.18(0.97-1.44) | 1.04(0.92-1.16) |
| Receiving oxygen on arrival | 1.65(1.56-1.75) | 1.83(1.72-1.94) | 0.76(0.64-0.90) | 1.07(0.69-1.66) | 2.23(1.85-2.69) |
| **Follow up visit to the ED vs. initial visit** | 0.74(0.70-0.78) | 0.61(0.57-0.65) | 1.29(1.16-1.42) | 1.38(1.04-1.82) | 0.77(0.62-0.96) |
|  |  |  |  |  |  |
| **Visited last 72 hours** | 0.76(0.71-0.82) | 0.71(0.66-0.77) | 0.99(0.86-1.14) | 1.41(1.01-1.96) | 0.59(0.44-0.79) |
| **Triage level** |  |  |  |  |  |
| Non-urgent | Reference |  |  |  |  |
| Immediate | 3.99(3.50-4.56) | 4.30(3.71-4.97) | 2.26(1.61-3.17) | 1.27(0.43-3.72) | 8.35(5.09-13.68) |
| Emergent | 4.34(3.99-4.72) | 4.70(4.27-5.17) | 2.57(2.11-3.13) | 2.44(1.48-4.03) | 7.12(4.82-10.52) |
| Urgent | 4.10(3.79-4.44) | 4.12(3.76-4.51) | 3.93(3.30-4.69) | 2.51(1.59-3.95) | 5.64(3.87-8.22) |
| Semi-urgent | 1.46(1.34-1.58) | 1.48(1.35-1.63) | 1.35(1.12-1.62) | 1.38(0.86-2.21) | 1.49(1.00-2.21) |
| **Visit related to an injury, poisoning, or adverse effect of medical treatment** | | | | | |
| No | Reference |  |  |  |  |
| Yes | 0.79(0.77-0.81) | 0.98(0.95-1.01) | 0.24(0.22-0.26) | 0.82(0.68-0.97) | 0.41(0.37-0.47) |
| **Is the injury/poisoning intentional** | | | | | |
| Not an injury/poisoning visit | Reference |  |  |  |  |
| Yes, self-inflicted | 0.47(0.40-0.55) | 0.61(0.52-0.71) | 0.15(0.08-0.26) | 0.13(0.02-0.93) | 0.08(0.02-0.32) |
| Yes, assault | 1.96(1.80-2.14) | 2.59(2.37-2.83) | 0.34(0.24-0.49) | 0.12(0.02-0.87) | 0.64(0.39-1.03) |
| No, unintentional | 0.80(0.78-0.83) | 1.01(0.98-1.05) | 0.19(0.16-0.21) | 0.87(0.72-1.06) | 0.46(0.40-0.53) |
| **Pain Scale** |  |  |  |  |  |
| 0-2 | Reference |  |  |  |  |
| 3-6 | 1.27(1.22-1.32) | 1.19(1.14-1.24) | 1.94(1.77-2.13) | 1.23(0.95-1.59) | 0.94(0.80-1.11) |
| 7 – 10 | 1.39(1.35-1.44) | 1.32(1.27-1.37) | 1.87(1.72-2.04) | 1.51(1.21-1.89) | 1.31(1.15-1.49) |
| **Cancer** | 1.52(1.39-1.66) | 1.72(1.56-1.89) | 0.62(0.47-0.83) | 1.52(0.90-2.56) | 1.58(1.14-2.18) |
| **Cerebrovascular Disease** | 2.43(2.29-2.58) | 2.64(2.48-2.82) | 0.81(0.66-0.98) | 2.75(1.99-3.80) | 4.47(3.80-5.26) |
| **Congestive heart failure** | 1.21(1.13-1.29) | 1.29(1.21-1.38) | 0.75(0.63-0.90) | 1.06(0.69-1.62) | 1.46(1.17-1.82) |
| **Chronic obstructive pulmonary disease** | 1.03(0.95-1.12) | 1.15(1.04-1.26) | 0.50(0.38-0.65) | 1.19(0.73-1.95) | 1.00(0.72-1.40) |
| **Dementia** | 2.55(2.24-2.90) | 2.99(2.61-3.41) | 0.83(0.54-1.27) | 1.19(0.44-3.20) | 2.22(1.40-3.54) |
| **Diabetes** | 1.24(1.19-1.29) | 1.31(1.25-1.37) | 0.85(0.77-0.93) | 1.38(1.09-1.74) | 1.50(1.30-1.71) |
| **Condition requiring dialysis** | 1.19(1.06-1.34) | 1.21(1.06-1.38) | 0.89(0.66-1.20) | 1.65(0.88-3.10) | 1.61(1.10-2.37) |
| **Pulmonary embolism** | 1.60(1.34-1.91) | 1.54(1.27-1.87) | 1.54(1.05-2.25) | 2.96(1.39-6.30) | 2.12(1.22-3.70) |
| **Myocardial infarction** | 1.31(1.20-1.44) | 1.44(1.31-1.59) | 0.60(0.46-0.79) | 1.29(0.75-2.21) | 1.75(1.30-2.35) |
| **HIV** | 0.75(0.63-0.88) | 0.78(0.65-0.94) | 0.48(0.30-0.77) | 0.95(0.36-2.55) | 0.95(0.52-1.72) |
