# Supplementary material for: Advanced diagnostic imaging utilization during emergency department visits in the United States: A predictive modeling study for emergency department triage
Source: PLoS One. 2019 Apr 9;14(4):e0214905. doi: 10.1371/journal.pone.0214905 (PMC6456195; doi:10.1371/journal.pone.0214905)
Supplement: S3 Table — (DOCX) [file pone.0214905.s004.docx]

**S3 Table**. Most frequent 10 words in each topic model for Topics 1-20

| **Topic 1** | **Topic 2** | **Topic 3** | **Topic 4** | **Topic 5** | **Topic 6** | **Topic 7** | **Topic 8** | **Topic 9** | **Topic 10** |
| --- | --- | --- | --- | --- | --- | --- | --- | --- | --- |
| foot | blood | breath | fall | cut | soreness | unspecified | symptom | cough | vomit |
| toe | test | shortness | other | laceration | ache | injury | toothache | congestion | nausea |
| ache | result | fluid | subsequent | facial | arm | multiple | unspecified | nasal | diarrhea |
| soreness | glucose | abnormality | striking | area | discomfort | site | edema | throat | associate |
| swell | vomit | aspiration | accident | neck | pain | decrease | refer | sinus | hiv |
| nail | stool | symptom | wrist | stiffness | pedal | appetite | gum | fever | condition |
| ingrown | melena | bodily | unspecified | limitation | accident | sclerosis | teeth | wheeze | without |
| accident | hematemesis | asthma | injury | trunk | cyclist | escalator | jaw | voice | temper |
| amputation | find | cortical | commode | ankle | cycle | ache | facial | hoarseness | blank |
| perform | radiological | flatulence | upper | earache | col | insomnia | dental | sneeze | commode |
|  |  |  |  |  |  |  |  |  |  |
| **Topic 11** | **Topic 12** | **Topic 13** | **Topic 14** | **Topic 15** | **Topic 16** | **Topic 17** | **Topic 18** | **Topic 19** | **Topic 20** |
| head | shortness | cramp | boil | unspecified | pain | pain | chest | pain | poison |
| upper | breath | abdominal | furuncle | accidental | chest | chest | pressure | chest | effect |
| respiratory | asthma | stomach | carbuncle | environmental | rib | hypertension | tightness | nutritional | drug |
| cold | wheeze | spasm | cellulitis | accident | abdominal | mellitus | arrival | endocrine | adverse |
| infection | care | esophagus | medicinal | wind | fever | diabetes | unconscious | meet | self |
| cough | patient | pain | substance | puncture | injection | arrest | discomfort | gland | suicide |
| flu | spokesperson | diarrhea | edema | site | knee | cardiac | sinus | enlarge | accidental |
| bronchitis | refuse | flatulence | drug | allergy | lung | fullness | paleness | agent | inflict |
| neck | groin | musculoskeletal | infectious | gunshot | testis | distention | pallor | thyroid | abuse |
| sound | edema | asthma | acne | hypodermic | antirheumatics | cardiopulmonary | doa | antidiabetic | late |
