# Supplementary material for: Advanced diagnostic imaging utilization during emergency department visits in the United States: A predictive modeling study for emergency department triage
Source: PLoS One. 2019 Apr 9;14(4):e0214905. doi: 10.1371/journal.pone.0214905 (PMC6456195; doi:10.1371/journal.pone.0214905)
Supplement: S4 Table — (DOCX) [file pone.0214905.s005.docx]

**S4 Table**. Parameter estimation between the outcome of ADI use and structured imaging during the emergency department visit, NHAMCS 2009-2014

|  | Coefficient | Standard Error | Standardized  Coefficient | p value |
| --- | --- | --- | --- | --- |
| **Age group** |  |  |  |  |
| 18-29 years | Reference |  |  |  |
| 30-44 years | -0.204 | 0.014 | -0.081 | <.0001 |
| 45-64 years | -0.082 | 0.013 | -0.033 | <.0001 |
| 65-74 years | 0.220 | 0.020 | 0.068 | <.0001 |
| ≥75 years | 0.371 | 0.019 | 0.121 | <.0001 |
|  |  |  |  |  |
| **Sex (male vs female)** | -0.083 | 0.014 | -0.023 | <.0001 |
| **Ethnicity (Hispanic vs Non-Hispanic)** | 0.085 | 0.020 | 0.016 | <.0001 |
| **Race** |  |  |  |  |
| White | Reference |  |  |  |
| African American | -0.167 | 0.015 | -0.077 | <.0001 |
| Others | 0.098 | 0.022 | 0.030 | <.0001 |
| **Residence** |  |  |  |  |
| Private residence | Reference |  |  |  |
| Nursing home | 0.069 | 0.039 | 0.013 | 0.081 |
| Homeless | -0.444 | 0.064 | -0.069 | <.0001 |
| Other | 0.205 | 0.046 | 0.034 | <.0001 |
| **Arrived by Ambulance** | 0.625 | 0.018 | 0.134 | <.0001 |
| **Source of payment** |  |  |  |  |
| Private Insurance | Reference |  |  |  |
| Medicare | -0.078 | 0.016 | -0.033 | <.0001 |
| Medicaid or CHIP | -0.065 | 0.016 | -0.025 | <.0001 |
| Uninsured | -0.060 | 0.017 | -0.022 | 0.000 |
| Other | 0.009 | 0.028 | 0.003 | 0.740 |
| **Day of Week** |  |  |  |  |
| Sunday | Reference |  |  |  |
| Monday | 0.011 | 0.016 | 0.003 | 0.473 |
| Tuesday | -0.001 | 0.016 | 0.000 | 0.951 |
| Wednesday | 0.008 | 0.016 | 0.002 | 0.632 |
| Thursday | 0.012 | 0.017 | 0.003 | 0.490 |
| Friday | 0.007 | 0.017 | 0.002 | 0.686 |
| Saturday | -0.026 | 0.017 | -0.007 | 0.125 |
| **Arrival time** |  |  |  |  |
| Morning | Reference |  |  |  |
| Afternoon | 0.015 | 0.009 | 0.008 | 0.104 |
| Evening | -0.047 | 0.012 | -0.018 | <.0001 |
| **Initial vital sign** |  |  |  |  |
| **Temperature** |  |  |  |  |
| 36 C-38 C | Reference |  |  |  |
| <36 C | 0.067 | 0.025 | 0.017 | 0.008 |
| >38 C | -0.102 | 0.035 | -0.019 | 0.003 |
| Pulse oximetry % |  |  |  |  |
| <=90 vs >90 (normal) | -0.276 | 0.024 | -0.050 | <.0001 |
| **Heart rate** |  |  |  |  |
| 60-100 | Reference |  |  |  |
| <60 | -0.007 | 0.018 | -0.002 | 0.704 |
| >100 | -0.002 | 0.014 | -0.001 | 0.891 |
| **Respiratory rate** | -0.004 | 0.002 | -0.010 | 0.012 |
| **DBP** |  |  |  |  |
| 60-80 | Reference |  |  |  |
| <60 | 0.005 | 0.018 | 0.002 | 0.796 |
| >80 | -0.004 | 0.013 | -0.002 | 0.740 |
| **SBP** |  |  |  |  |
| 80-120 | Reference |  |  |  |
| <80 | -0.192 | 0.035 | -0.052 | <.0001 |
| >120 | 0.102 | 0.020 | 0.049 | <.0001 |
| Receiving oxygen on arrival | -0.076 | 0.033 | -0.008 | 0.019 |
| **Follow up visit to the ED vs. initial visit** | -0.133 | 0.032 | -0.018 | <.0001 |
| **Visited last 72 hours** | -0.163 | 0.039 | -0.018 | <.0001 |
| **Triage level** |  |  |  |  |
| Non-urgent | Reference |  |  |  |
| Immediate | 0.399 | 0.047 | 0.057 | <.0001 |
| Emergent | 0.479 | 0.021 | 0.104 | <.0001 |
| Urgent | 0.367 | 0.016 | 0.122 | <.0001 |
| Semi-urgent | -0.476 | 0.020 | -0.140 | <.0001 |
| **Visit related to an injury, poisoning, or adverse effect of medical treatment** | | | | |
| No | Reference |  |  |  |
| Yes | -0.496 | 0.030 | -0.129 | <.0001 |
| **Is the injury/poisoning intentional** | | | | |
| Not an injury/poisoning visit | Reference |  |  |  |
| Yes, self-inflicted | -0.640 | 0.061 | -0.166 | <.0001 |
| Yes, assault | 1.010 | 0.041 | 0.270 | <.0001 |
| No, unintentional | -0.032 | 0.027 | -0.015 | 0.234 |
| **Pain Scale** |  |  |  |  |
| 0-2 | Reference |  |  |  |
| 6-Mar | 0.018 | 0.009 | 0.008 | 0.057 |
| 7 – 10 | 0.302 | 0.010 | 0.125 | <.0001 |
| **Cancer** | 0.110 | 0.048 | 0.008 | 0.021 |
| **Cerebrovascular Disease** | 0.551 | 0.033 | 0.055 | <.0001 |
| **Congestive heart failure** | -0.300 | 0.036 | -0.031 | <.0001 |
| **Chronic obstructive pulmonary disease** | -0.300 | 0.046 | -0.025 | <.0001 |
| **Dementia** | 0.416 | 0.070 | 0.019 | <.0001 |
| **Diabetes** | -0.041 | 0.022 | -0.007 | 0.057 |
| **Condition requiring dialysis** | -0.079 | 0.063 | -0.005 | 0.208 |
| **Pulmonary embolism** | 0.176 | 0.093 | 0.006 | 0.060 |
| **Myocardial infarction** | -0.122 | 0.049 | -0.009 | 0.012 |
| **HIV** | -0.197 | 0.089 | -0.009 | 0.028 |
